# Anatomical 3D-Printed Silicone Prostate Gland Models and Rectal Examination Task Trainer for the Training of Medical Residents and Undergraduate Medical Students

**DOI:** 10.7759/cureus.9020

**Published:** 2020-07-06

**Authors:** Jasmine DeZeeuw, Noel B O'Regan, Christine Goudie, Michael Organ, Adam Dubrowski

**Affiliations:** 1 Faculty of Medicine, Memorial University of Newfoundland, St. John's, CAN; 2 Anesthesiology, Memorial University of Newfoundland/Janeway, St. John's, CAN; 3 Medical Education and Simulation, Memorial University of Newfoundland, St. John's, CAN; 4 Urology, Memorial University of Newfoundland, St. John's, CAN; 5 Health Sciences, Ontario Tech University, Oshawa, CAN

**Keywords:** simulation in medical education, digital rectal examination, urology, three-dimensional (3d) printing, task-trainer, prostate

## Abstract

The current generation of graduating medical students is entering into practice with minimal exposure to the digital rectal examination (DRE), a necessary component of a complete physical examination. Simulation-based medical education (SBME) using anatomical silicone models and task trainers can provide hands-on training opportunities for medical students to rehearse DREs. However, there is a scarcity of affordable, validated, and anatomically correct silicone prostate models and task trainers for rehearsing DREs. This technical report describes and validates evidence for silicone prostate models and a DRE task trainer created from three-dimensional (3D)-printed molds for medical student- and resident-training and clinical skills maintenance.

A pre-existing 3D human model and five different prostate models from open-source, royalty-free websites were converted using Fusion360™ (Autodesk Inc., San Rafael, CA) into stereolithography files and altered to produce negative molds. The prostate molds were filled with silicone and polylactic acid filament “nodules”. The buttocks were isolated from the human model and an anal canal was designed with a larger cavity on the interior to hold the silicone prostate models to simulate a real DRE. Five practicing urologists were recruited to evaluate the 3D-printed silicone prostate models and the DRE task trainer. The participants were provided with a qualitative survey and asked to rate the perceived realism and educational effectiveness of the prostate models and task trainer.

The silicone models and task trainer were found to be useful for simulation training when attempting DRE techniques. The feedback from the participants was positive overall and provided recommendations for improvement including stabilizing the prostate models in the task trainer, smoothening the transition between the rectum and the prostate, and adding an additional “normal” prostate model.

Silicone prostate models and DRE task trainers created from 3D molds are economical and anatomically and tactically accurate training tools to teach and maintain DRE skills as compared to commercially available, cost-prohibitive models. After making the suggested and appropriate modifications, the prostate models and DRE task trainer could potentially be used as tools for clinical skills training and maintenance and for patient education in the future.

## Introduction

Prostate cancer is the most frequently diagnosed cancer among men in Canada, with over 21,000 new cases diagnosed in 2017; and it is directly linked to a 10% fatality rate [1]. Screening for prostate cancer is commonly done through a prostate-specific antigen (PSA) test with or without a digital rectal examination (DRE) [2]. However, there is a need for improved simulation-based training methods at the pre-clerkship, clerkship, and resident levels for more accurate identification of prostate pathologies within rectal examinations. DREs are commonly performed by urologists to screen for a variety of prostate pathologies, including prostate enlargement, infection, and cancer [3,4]. The DRE involves a physician inserting a finger into the rectum and then combining tactile clues with knowledge of the underlying anatomy and clinical history to make an informed diagnosis [5]. Although the DRE is potentially indicated in clinical settings spanning the fields of family medicine, urology, gastroenterology, and trauma care, it is not widely utilized due to the reported low confidence and comfort level of physicians in performing a DRE [3,4,6,7]. In a recent survey of Canadian medical schools, it was found that 62% of pre-clerkship students performed less than two DREs throughout their whole training period and that there are generally no formal DRE teaching sessions during clerkship [7]. This finding reveals a need for increased hands-on training opportunities for medical students relating to DRE.

Prostate cancer screening is one of the most controversial areas in urology. The Canadian Task Force on Preventive Health Care does not recommend screening for prostate cancer with the PSA blood test or the DRE in men without a previous diagnosis of prostate cancer [8]. However, the Canadian Urological Association has recommended that family practitioners start offering PSA testing at 50 years of age for most men and at 45 years of age for men with a high risk of prostate cancer [9]. Men electing for PSA screening are often offered a DRE for additive information beyond the PSA [9]. In the event that a patient has an elevated PSA and/or abnormal DRE findings, the patient should be referred to a urologist. Additionally, the Canadian Urological Association guidelines have listed the DRE as a mandatory part of the workup for lower urinary tract symptoms in male patients, which affect 28% of men over the age of 70 [10,11]. When used correctly and judiciously, the DRE can be an essential physical examination skill that all medical graduates should be proficient in.

Because of the invasive nature and lack of visual inspection scope, the DRE is a challenging skill to teach [6]. The DRE is becoming increasingly understood and implemented through advanced simulation-based medical education (SBME), but there are concerns about the lack of commercially available products to assist with such training, especially ones that accurately simulate the tactile properties of human tissue and abnormalities [6]. There is definitely a need to augment DRE training through accurate simulations of the tactile properties and abnormalities of the human prostate.

SBME provides a safe, controlled environment for medical trainees to practice DRE competencies. SBME has advanced significantly in the past two decades with the help of three-dimensional (3D) printing as a way to produce anatomical models and simulation tools [12]. Such haptic, anatomically correct 3D-printed models provide opportunities to help with the understanding of specific medical conditions and pathologies [13]. Simulation training is thought to be an optimal way for physicians in fields such as family medicine and urology to gain psychomotor skills prior to patient contact, and specifically prior to examining the male prostate during DREs [14].

Prostate simulators that are currently in use lack or misrepresent tactile cues, present a limited number of scenarios, and offer little by way of feedback to students. Such simulators include the Life/form® Prostate Examination Simulator (Nasco, Fort Atkinson, WI) and the G300 life-size prostate model set (Anatomical Chart Company, Skokie, IL). The idea of using 3D prostate models to train physicians on how to identify pathologies during a DRE predates the past decade. In 2000, Yanoshak et al. published a research article that argued that family physicians who trained with a 3D prostate model found a stronger agreement between transrectal ultrasound and DRE prostate size estimates [15]. In the past 10 years, the research and technology for developing prostate models and simulations have advanced tremendously. Qiu et al. developed prostate simulators using 3D-printed prostate models to mimic the physical properties of the tissue and integrated soft electronic sensors using custom-formulated polymeric inks as a surgical aid for preoperative planning and rehearsal [16]. The simulators thus developed demonstrated high fidelity with the patient prostate gland and tissue properties (anatomical, mechanical, tactile, and optical) to predict organ physical behaviour more accurately [16]. Kowalik et al. developed a prostate simulator using synthetic elastomers and expandable balloons [5]. The models were an attempt to authenticate the accuracy of the simulated prostate elasticity to the range of normal prostate stiffness, to determine the range of nodule size reasonably palpable by DRE, and to determine the degree of elasticity difference within the same prostate, which is suggestive of potential malignancy [5]. The results of their research concluded that the relationship between the nodule and the background prostate elasticity constitutes the critical tactile feedback from a DRE suggesting a pathology, not the absolute elasticity of the nodule [5]. Hence, they recommend that DREs should be a part of the educational curriculum as they can be used to diagnose adenocarcinoma from palpating abnormalities on the prostate [5]. Although these studies did not validate the efficacy of the prostate simulation in an educational setting, the urologists in the study confirmed that the stiffness values of the prostate models reflected what is actually palpated in practice [5]. Thus, it is clear that the literature perceives 3D prostate models as effective training tools for the DRE.

The purpose of this technical report is to describe the process of designing, manufacturing, and validating novel 3D-printed silicone prostate models and a DRE task trainer to be used for teaching prostate abnormality screening skills to students and residents. The validation of this model followed a quality improvement framework, involved expert clinicians with experience in the prostate examination, and focused on face validity (i.e. how closely do these models represent reality) and content validity (i.e. their perceived utility as training tools for family physicians and urologists).

## Technical report

Context

A survey of 13 Canadian medical schools has indicated that the DRE is taught to pre-clerkship students using various combinations of methods [7]. The results showed a mean of approximately 2.15 hours of pre-clerkship DRE training with most schools providing one formal DRE teaching session, no formal DRE training during clerkship, and no formal DRE evaluation [7]. A multifaceted approach delivers the most effective DRE teaching by using a wide variety of techniques including models, videos, physician instruction, and standardized patients [7]. Such an approach addresses all aspects of the DRE including technical skills, theoretical knowledge, feedback, interpersonal communication skills, and comfort in performing the examination. Residents and students would potentially benefit from anatomically accurate, affordable, and high-fidelity 3D-printed prostate models and a task trainer to help in technical training to perform DREs.

The DRE is routinely performed in urology clinics to evaluate the prostate gland [3,4]. Due to the high volume of DREs performed by urologists, five practicing urologists working at the Health Sciences Centre in St. John’s, Newfoundland were recruited to evaluate the 3D-printed silicone prostate models and the DRE task trainer. The silicone prostates and the DRE task trainer served as the first iteration prototypes for training and feedback purposes. One-on-one meetings between a facilitator and the participants were scheduled to evaluate and discuss the silicone models and the DRE task trainer.

Input(s)

**Figure 1:**
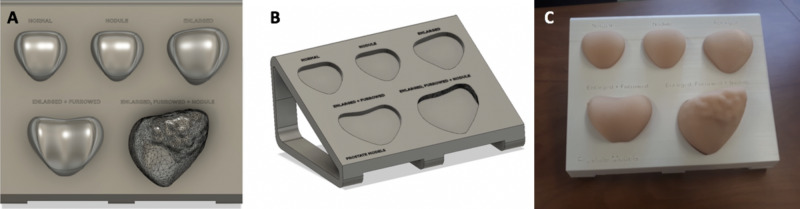
Silicon prostate models and base A: silicone prostate model designs; B: prostate model base design; C: silicone prostate models from 3D-printed molds

Five prostates of different shapes and their negative molds were designed into a stereolithography (.stl) file in Fusion360™ (Autodesk Inc., San Rafael, CA) based on pictures found on an open-source, royalty-free website (https://gpianatomicals.com) [17]. The prostates designed were: “normal”, “nodular”, “enlarged”, “enlarged and furrowed”, and “enlarged, furrowed, and nodular” (Figure 1). Using a secure digital (SD) card, the 3D-rendered prostate molds in the form of a .stl file were transferred to an Ultimaker 2 3D printer (Ultimaker B.V., Utrecht, Netherlands) and were printed using polylactic acid (PLA) filament material. The prostate models were made by filling the molds with the silicone mix EcoFlex 00-30 Platinum Cure Silicone Rubber (Smooth-On Inc., Macungie, PA) for the “normal”, “nodular”, and “enlarged” prostate models; Dragon Skin™ was used for the remaining two prostate models. The two prostate models with nodules had a PLA nodule inserted into them during the silicone filling.

A stand for the prostate models was designed using Fusion360™ so that the silicone prostates could friction-fit into the base (Figure 1). The inclined face of the stand was at an angle of 35 degrees and above each base was a label corresponding to each silicone prostate model. The stand .stl file was also transferred to an Ultimaker 2 3D printer and printed using white PLA filament material.

**Figure 2:**
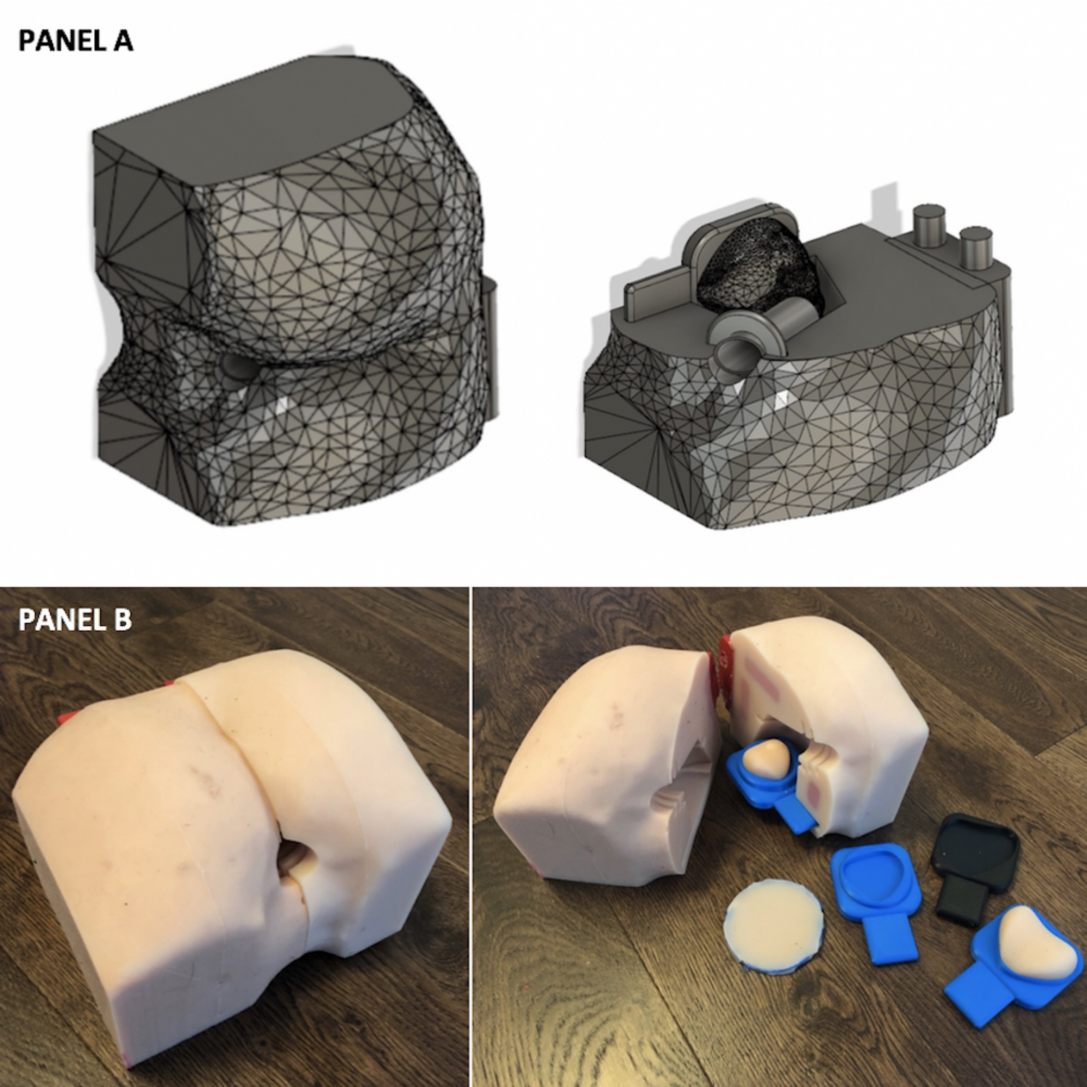
DRE task trainer Panel A: digital 3D model. Panel B: silicone DRE task trainer, prostate inserts, and skin flap membrane DRE: digital rectal examination

A silicone DRE task trainer and its negative mold were designed to allow for the tactile sensation of inserted silicone prostates without a visual aid, thereby simulating a real DRE. A pre-existing 3D human model was obtained from an open-source website and the buttocks were isolated. An anal canal was designed with a larger cavity on the interior to hold the silicone prostate models. Due to the size constraints of the 3D printers, each cheek of the buttocks was separated and holes with adjacent PLA pegs were added to attach the cheeks together. The mold was designed to leave voids in the silicone for the insertion of printed materials (prostate, sphincter muscles, etc.) (Figure 2). To insert a silicone prostate model, a flat PLA base for each of the five prostate models was developed. A silicone membrane was also designed to place over the prostate to simulate rectoprostatic fascia separating the prostate from the rectum.

Process

In addition to the silicone prostate models and the DRE task trainer, upon receiving verbal consent from the urologists, the facilitator provided a survey to determine the efficacy of the silicone models as a training tool for students (Table 1).

**Table 1:** Survey to evaluate the performance of the silicone prostate models and the DRE task trainer DRE: digital rectal examination

Evaluation of Silicone Prostate Models and Silicone DRE Task Trainer for the Rehearsal of Prostate Exams					
Instruction: Please rate the following ten questions as: 1 = Strongly disagree; 2 = Disagree; 3 = Somewhat agree; 4 = Agree; 5 = Strongly agree					
Evaluation of the Silicone Prostate Models	1	2	3	4	5
1. The flexibility of the prostate models are appropriate for training					
2. The tactile textures of the prostate models are appropriate for training					
3. The sizes of the prostate models are appropriate for training					
4. The colours of the prostate models are appropriate for training					
5. The anatomical features seen on the prostate models are appropriate for training					
6. Each silicone prostate model is identifiable in shape and texture when placed inside the DRE task trainer for simulation purposes					
Evaluation of the Silicone DRE Task Trainer	1	2	3	4	5
7. Using the silicone DRE task trainer model will help to increase the trainees' competency in performing prostate exams					
8. Using the silicone DRE task trainer will help increase the trainees' confidence					
9. Witnessing the trainees' performance on this silicone DRE task trainer will increase my confidence in their ability to assist in the clinic					
10. Witnessing the trainees' performance on this silicone DRE task trainer will increase my confidence in their ability to perform the skills trained on the model in the clinic					
11. The silicone DRE task trainer would be a valuable addition to current simulation-based medical education					
12. What revisions, if any, would you suggest to improve the functionality of the silicone DRE task trainer?					

The survey included 12 structured questions, with 11 questions prompting participants to score between 1-5 on a linear Likert scale and one open-ended question prompting feedback about suggested improvements. The survey was designed to elicit participant feedback regarding the face and content validity of the silicone prostate models and the DRE task trainer. Mean and standard deviations were determined for the evaluation scores from the entire cohort. If the mean question response was less than 3.5 out of 5 and the standard deviation was greater than 1, a review of the comments and revisions to the models and/or task trainer would be warranted.

Products/outcomes

**Figure 3:**
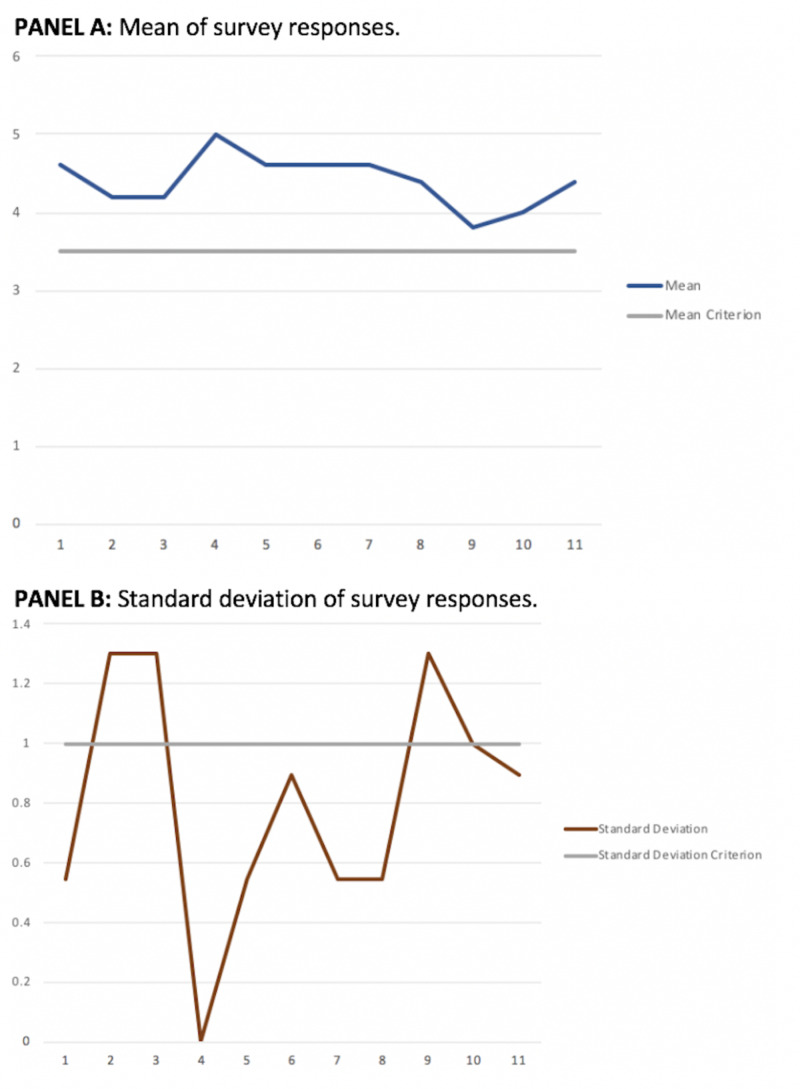
Mean and standard deviation of survey questions Panel A: mean of survey responses. Y-axis: mean; X-axis: survey question as related to each number respectively in Table 1 Panel B: standard deviation of survey responses. Y-axis: standard deviation; X-axis: survey question as related to each number respectively in Table 1

On average, participants gave a rating of 4.53 out of 5 to the silicone prostate model portion, and 4.24 out of 5 to the DRE task trainer portion with regard to their appropriateness and usefulness in education. The average rating for the value of adding the silicone DRE task trainer to current medical education was 4.4 out of 5. All answers were above the criterion for reevaluation and all participants provided constructive feedback for improvements for subsequent iterations of the models and task trainer (Figure 3).

The standard deviations to three of the questions were greater than 1, indicating that there was a degree of disagreement among the participants. Therefore, we followed up with a free text questionnaire to investigate the reasons for the disagreement.

Question nine had a mean response close to the criterion at 3.8 out of 5 and a standard deviation of 1.3. The question was aimed at determining if participants would be more confident in the ability of students to assist with the DRE after performing the maneuver on the task trainer. There was one outlier, and on follow-up, the urologist who had chosen a low score for this question explained that there was no better way to learn the DRE than to perform the examination on patients. However, he also stated that the task trainer is a good tool to introduce students to the topic and technique to perform the DRE.

Questions two (mean: 4.2, SD: 1.3) and three (mean: 4.2, SD: 1.3), which dealt with the tactile texture and size of the prostate models, also had standard deviations greater than 1. Upon further investigation, it was discovered that one urologist felt that the silicone model of the “normal” prostate was too small and that the model of the “enlarged” prostate was also too small for benign prostatic hyperplasia. In discussions with two other urologists, they stated that the “normal” prostate model size was appropriate for a young, healthy male. As the majority of DREs performed in the urology practice is done on middle-aged to elderly men, often with abnormal findings on DRE, the prostates examined by urologists are often larger than the size of the model. One urologist mentioned that some patients have a naturally flatter prostate than the model and suggested that an additional “normal” prostate model should be added for such prostates. One element that was common among the comments on the tactile texture of the models was the perception that the silicone model of the “normal “prostate and the nodule in the “enlarged, furrowed, and nodular” prostate model were not firm enough. One urologist compared the firmness of a normal prostate to a flexed thenar eminence and the nodule to a thumb knuckle.

## Discussion

The silicone prostate models and DRE task trainer were validated by study participants as a useful, anatomically correct simulation tool for DRE training of medical students and residents. The response to the first iteration of the models and task trainer was positive overall and provided a number of suggestions to make the models more accurate and the task trainer fully functional as a simulation tool to be integrated into an SBME learning curriculum. Although the participants were impressed by the DRE task trainer as a whole, they had several suggestions to offer for improvement. Two of the five urologists mentioned that the prostates placed in the DRE task trainer were not stable enough to sweep their fingers across to feel for the prostate margins and changes in consistency. Another comment was that the prostate placement inside the simulated rectum was deeper than the one found in most patients while being appropriate enough for overweight patients. Additionally, one participant noticed that upon entry into the rectum, there was an abrupt drop between the perineum and the prostate model rather than a smooth membrane, the rectoprostatic fascia, overlying the prostate. The prototype for such a membrane was shown to the urologists. There were mixed opinions on whether a membrane was a necessary addition to the task trainer; however, all agreed that the prototype membrane was at least three times the thickness of real rectoprostatic fascia. Based on the feedback obtained, future iterations of the task trainer should include stabilization of the prostate within the task trainer and a smoother transition between the rectum and the prostate. Future iterations of the silicone prostate models should have the “normal” prostate model slightly firmer, by making the nodule in the “enlarged, furrowed, nodular” prostate model firmer or bringing the PLA nodule closer to the surface of the silicone prostate; also adding a second, more flattened “normal” prostate model should be considered.

The urologists recruited for this study were impressed by the quality and accuracy of the silicone prostate models. The participants stated that the models would be a good addition to DRE training of medical students and residents in combination with teaching and practicing on standardized or real patients. Although prostate models and DRE simulations are already available, the 3D-printed prostate models and DRE task trainer are predictably less expensive, yet equally or more effective for training purposes based on the survey results.

Two of the participants also suggested that nurse practitioners should also be recruited to evaluate the models as they are part of the urology team at the Health Sciences Centre and often perform the DRE before the urologists. As such, nurse practitioner students may also benefit from the prostate models and DRE task trainer in addition to medical students. Additionally, the DRE is an invasive examination that may be beneficial in the diagnosis of prostate pathologies; but due to the nature of the examination, some patients choose not to have one performed. The prostate models and DRE task trainer may be effective tools for patient education to communicate the purpose of the examination and to clarify any misconceptions.

One limitation regarding the validation of the silicone prostate models and DRE task trainer was that the results were based on the feedback of only five participants in the same department of the Health Sciences Centre. However, data saturation was reached. Further investigations should include inputs from family physicians and nurse practitioners with experience in performing the DRE.

## Conclusions

Silicone prostate models and DRE task trainers created from 3D molds are a cost-effective method to accurately teach and maintain DRE skills. This technical report described the development of 3D-printed prostate models and a task trainer for DRE simulation and the evaluation of these components with respect to DRE teaching. The results of the research survey completed by practicing urologists revealed that the silicone models and task trainer would be valuable additions to DRE training. The urologists suggested some modifications that should be made for future iterations of the models and task trainer to improve on the anatomical and tactical realism of the tools. After making the suggested and appropriate modifications, the prostate models and DRE task trainer could potentially be used as tools within SBME, clinical skills training and maintenance, and for patient education in the future.
